# Genome-wide association study reveals heat tolerance QTL for canopy-closure and early flowering in chickpea

**DOI:** 10.3389/fpls.2024.1458250

**Published:** 2024-12-17

**Authors:** Cara Jeffrey, Brent Kaiser, Richard Trethowan, Laura Ziems

**Affiliations:** ^1^ School of Life and Environmental Sciences, The University of Sydney, Sydney, NSW, Australia; ^2^ The Sydney Institute of Agriculture, The University of Sydney, Sydney, NSW, Australia; ^3^ The Plant Breeding Institute, The University of Sydney, Sydney, NSW, Australia

**Keywords:** *Cicer arietinum*, heat stress, abiotic trait, GWAS, quantitative genetics

## Abstract

Chickpeas are a vital source of protein and starch for a large portion of the world’s population and are known to be impacted by heat stress at every life stage. Previously known as an “Orphan Legume”, little is known of the genetic control of heat stress tolerance, and most previous research has focused on heat avoidance rather than tolerance. This study utilised a population of 148 chickpea genotypes, primarily Kabulis, in 12 field trials conducted at 2 locations, two sowing periods, and across 3 years. Physiology was examined, and data was paired with Diversity Arrays Technology (DArT) sequencing to perform a Genome Wide Association Study to connect phenotypic and genotypic regions. Fourteen QTL related to yield, seed size, time to flowering, time to maturity, and final canopy closure were found. Among these, are the first Quantitative Trait Loci (QTL) ever identified for canopy closure in chickpea, along with a QTL that is likely linked to early flowering under heat stress. Early flowering in this case refers to a cultivar flowering significantly earlier than the others in the genotype set. Additionally, several other QTL provide validation of previous research. These QTL hotspots that can be targeted for selective breeding of several traits concurrently. Overall, new targets for genome assisted breeding for heat tolerance in chickpea were identified and can be utilised by the breeder community to improve the status of selective breeding for heat tolerance in this crop.

## 1 Introduction

Chickpea (*Cicer arietinum*) is a crucial source of protein and starch in many countries (Abbo et al., 2003), making up roughly 20% of global pulse production (FAO, 2020). Chickpeas are a nutrient dense food, with high protein, iron, and starch contents, and are an agronomically strong crop, surviving on low nutrient soils through highly efficient nitrogen extraction via symbiosis with rhizobial bacteria (Sharma et al., 2013). Heat stress impacts every stage of plant growth and development (Jeffrey et al., 2021) and the latest Intergovernmental Panel on Climate Change (IPCC) report has found that between 2021-2040 it is very likely that the global surface temperature will increase by 1.5 °C relative to 1850-1900 (IPCC, 2021). It has been shown that high temperatures can decrease potential yield of this crop by 10-15% for every degree that temperature increases beyond the minimum stress level (Upadhyaya et al., 2011), with extreme heat events leading to a complete loss of yield (Kumar et al., 2013). With the increased urgency of mitigating the effect of climate change on global cropping systems, the last two decades have seen an increased focus on breeding for heat and drought tolerance in chickpea (Devasirvatham et al., 2015; Maphosa et al., 2020).

One of the main issues in chickpea genomic research is the relative paucity of sequence information compared to other crops such as wheat (Alonge et al., 2020), even though the chickpea genome is small at roughly 12% that of wheat (Varshney et al., 2017). In the last decade there has been a surge in research linking genetic markers to traits of interest driven by advancements in genotypic technologies (Thudi et al., 2014; Farahani et al., 2019). High density genotyping including over 10,000 SNP markers has been accessible since 2012 which has expediated genetic research in chickpea (Hiremath et al., 2012). This technology has been utilised in a number of studies to further understand genetic diversity, linkage disequilibrium and allelic diversity, essential for chickpea improvement (Thudi et al., 2014; Roorkiwal et al., 2016; Farahani et al., 2019). The most recent genome-wide marker map was produced using Single-Nucleotide Polymorphisms (SNPs) in 2013 (Varshney et al., 2013) with the previous iteration produced with microsatellite Simple Sequence Repeats (SSRs) in 2000 (Winter et al., 2000). Because of this, there is significant inconsistency in the markers used in research since 2000, making it difficult to understand the locations of previously reported marker trait associations (MTA) and quantitative trait loci (QTL).

All *Cicer* species are diploid, with 16 somatic chromosomes, but there are sufficient differences between chromosome length and chromosomal constrictions to distinguish *C. arietinum* from the wild progenitor *C. reticulatum*, while maintaining a large degree of intraspecific variation in karyotype (Varshney et al., 2017). Due to this speciation, early attempts to form heterotic hybrids have been mostly unsuccessful, due to post fertilisation abortion (Ahmad and Slinkard, 2004). As such, the primary direction for crop improvement via genomic technologies seems to be improvement of existing map resources, with the goal of developing enhanced genomic tools to assist breeding (GAB) programs (Pandey et al., 2016).

With the accelerating impacts of climate change, improved understanding of the chickpea genome and validated MTAs for GAB programs, the genetics underlying the physiological response of chickpea to heat stress needs to be further investigated. There have been markers found for traits that are known to be linked to performance under heat stress, such as flowering time, seed size, and plant height (Kumar and Abbo, 2001; Thudi et al., 2014). However, the extent of this research is limited, and many of these studies have been performed on only a small subset of genotypes, missing the full diversity of physiological responses possible in the crop.

This study used 148 chickpea genotypes in two commercial growing regions in Australia to assess physiological traits under typical and heat-stressed conditions. With these physiological trait data, a Genome-Wide Association Study (GWAS) was completed. The aim was to identify key QTL associated with traits related to or impacted by heat stress, to advance future genetic improvement and research into heat tolerance in this key crop. Additionally, the findings of previous research were combined to produce a chromosome map showing key genes and QTL associated with heat stress and similar physiological traits. The objective was to create a simplified resource that depicts known genomic regions of interest, regardless of the marker type that was used to find them.

## 2 Materials and methods

### 2.1 Source of germplasm

Germplasm from an international genebank, consisting of 148 genotypes were examined, comprising 10 desi and 137 kabuli types. These genotypes originated from Australia (9), ICARDA (129), and ICRISAT (9), and are a mix of released cultivars and elite breeding lines (**Appendix 1**). These genotypes were selected to represent a diverse range of responses to heat stress.

### 2.2 Field trials

In total, data was collected across 3 years, 2 locations, and 2 sowing times, resulting in 12 field trials. Field trials were conducted in Kununurra, Western Australia (15.7783° S, 128.7439° E), and Narrabri, New South Wales (30.2737° S, 149.7350° E), using agronomic management typical of each region. Thus, management practices were not the same at each location. These locations were selected to represent the historical mediterranean environment used for cultivating chickpeas (Narrabri) and a higher temperature environment (Kununurra). Genotypes were sown according to commercial standards (~169 seeds and ~381 seeds per plot in Narrabri and Kununurra respectively) in randomised complete block designs of 2 replicates for each time of sowing (TOS) in plots of 8 m^2^. Trials in Kununurra were flood-irrigated fortnightly, with regular Nitrogen side-dressing throughout the season, whereas Narrabri trials were irrigated to simulate a typical year, inoculated with rhizobium and a pre-emergent herbicide applied. Temperature was collected in hourly intervals at each location, and daily minima and maxima has been provided in **Appendix 2**. These data can be found in raw form through weather.agric.wa.gov.au (Kununurra site) and ozforcast.com.au (Narrabri site). Delayed sowing trials were carried out annually between 2018-2020 inclusive, with the first sowing (TOS1) corresponding to the regional commercial season, and the second (TOS2) delayed by 1 month in Kununurra, and 2 in Narrabri. This variation in sowing delay between the regions is due to the shorter season length in Kununurra, caused by consistently higher temperatures than those of Narrabri (**Appendix 2**).

### 2.3 Measured traits and data curation

For all 3 years, sowing times and locations, the following traits were measured: Gross yield per plot (grams), hundred seed weight (HSW) (grams), flowering time, and maturity time. Flowering and maturity time were measured via a count of the number of days between sowing and the reaching of this set lifestage, as described in ICRISAT guidelines (IBPGR, ICRISAT, 1993). In addition, canopy closure was measured in the 2019 Narrabri trials using the “canopeo” app (Patrignani and Ochsner, 2015). Canopy closure measurements were taken towards the end of the podding phase, in order to ascertain the maximum canopy closure as a percentage of ground cover. To assess flowering and maturity time, plots were inspected as regularly as possible, typically every 2-3 days, to determine the date at which 50% of the plants in each plot had at least 1 flower and the date at which 50% of each plot was desiccated and a brown colour. These dates were then used to calculate the number of days from sowing to flowering and maturity (Pundir et al., 1988).

Phenotypic data is summarised in **Table 1**. The predicted means were calculated using linear mixed models in Genstat software (VSN-International, 2022), and subsequent data was organised into Quartile-Quartile (QQ) plots in R, grouped by location and year (**Appendix 3**). Year, genotypes, and sowing dates were arranged as fixed effects for each location, with range, and row within reps and sowing dates within years arranged as random effects. This was done to determine data conformity to a normal distribution, to assess quality for GWAS analysis.

**Table 1:** Mean and standard deviation of examined traits across trials; year, TOS and site.

			Kununurra			Narrabri		
		Year	2018	2019	2020	2018	2019	2020
Gross Yield (g)	TOS1	Mean	2015.72 ± 39.01	2704.67 ± 45.38	2568.22 ± 57.19	2179.44 ± 29.46	1724.68 ± 23.95	978.53 ± 15.36
		SD	496.45	564.9	693.29	353.51	293.23	186.79
	TOS2	Mean	2133.07 ± 37.99	2917.73 ± 47.13	2073.07 ± 52.24	1404.42 ± 15.15	1165.58 ± 18.03	516.74 ± 18.02
		SD	483.43	586.68	633.27	187.35	220.04	219.14
HSW (g)	TOS1	Mean	35.64 ± 0.57	36.51 ± 0.57	33.44 ± 0.49	37.21 ± 0.56	35.16 ± 0.45	37 ± 0.49
		SD	7.14	7.00	5.86	6.66	5.46	5.87
	TOS2	Mean	33.78 ± 0.54	38.81 ± 0.6	31.24 ± 0.47	34.65 ± 0.5	31.57 ± 0.44	32.32 ± 0.43
		SD	6.78	7.38	5.60	6.10	5.30	5.14
Flowering Age (days)	TOS1	Mean	52.13 ± 0.45	57.98 ± 0.76	67.45 ± 0.55	87.78 ± 0.25	84.91 ± 0.44	97.49 ± 0.28
		SD	5.64	9.36	6.63	2.99	5.32	3.32
	TOS2	Mean	55.22 ± 0.49	52.49 ± 0.55	62.93 ± 0.25	78.18 ± 0.55	65.17 ± 0.28	67.23 ± 0.27
		SD	6.14	6.79	2.99	6.79	3.32	3.24
Maturity Age (days)	TOS1	Mean	111.03 ± 0.37	127.55 ± 0.26		129.3 ± 0.28	133.62 ± 0.2	153.68 ± 0.31
		SD	4.65	3.24		3.30	2.45	3.68
	TOS2	Mean	99.39 ± 0.25	133.39 ± 0.47		116.04 ± 0.25	102.34 ± 0.2	110.32 ± 0.35
		SD	3.16	5.82		3.09	2.39	4.21
Final Canopy Closure (%)	TOS1	Mean						79.7 ± 0.67
		SD						8.09
	TOS2	Mean						62.09 ± 0.45
		SD						5.41

### 2.4 DNA extraction

Seeds were sprouted in petri dishes until large enough to produce sufficient leaves for extraction. The first 3-4 young leaves per individual were collected and dried in an Eppendorf tube with silica gel for 2-3 days. Two ball bearings were added to each tube, and tissue was subsequently crushed with a lyser for 2min at 20 rpm. Ball bearings were then removed, and 800 µL of CTAB buffer added to each tube, with samples incubated at 65°C for 30-40 min, followed by 5 min at room temperature. 600 µL Phenol-chloroform (24:1 ratio) was added to each tube, and samples were mixed for 2 min and then centrifuged at 10,000 rpm for 10 min. 600 µL supernatant was removed and placed in a 1.5 mL microcentrifuge tube, followed by 500 µL cold isopropanol. Samples were then chilled at -20°C for 20 min, and then centrifuged at 10,000 rpm for 10 min. Supernatant was discarded, and remaining ethanol was removed through drying. The DNA pellet was washed via the addition of 500 µL washing to each tube, and the use of a centrifuge at 7,000 rpm for 10 min. Samples were then dried, and 100 µL TE (pH 8) and RNAse (1:100 ratio) was added to each tube, before incubation at 37°C for 1-2 hours. DNA samples were diluted with double-distilled autoclaved water to 50 ng/µL and stored at -20°C until use.

### 2.5 Marker filtering

DNA samples were sent for DArT-SeqTM genotyping through Diversity Arrays technologies Pty Ltd (Canberra, Australia), which returned 3,339 SNP markers. Data was curated by removing poor-quality markers. Markers with minor allele frequency < 5%, markers that failed to provide information for > 20% of lines, and markers without mapped positions on the *Cicer arietinum* CDC Frontier genome v1.0 map (BioProject PRJNA175619) (Varshney et al., 2013) were removed. 2,361 reported SNPs and 1,919 mapped positions were used for the GWAS analysis.

### 2.6 Genetic population structure

**Figure 1:**
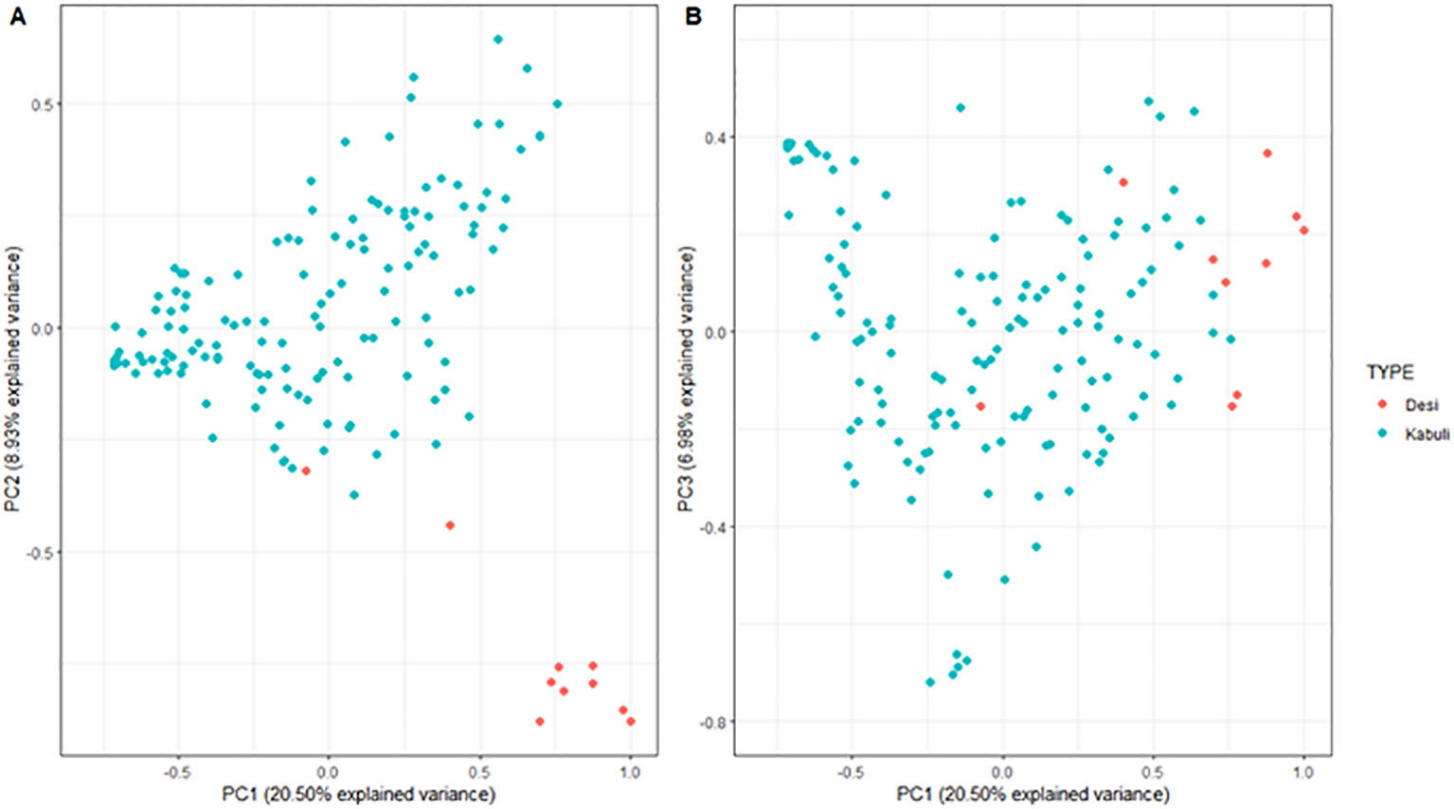
Principal component analysis (PCA) of the kinship matrix visualizing the genetic relationships between147 desi and kabuli chickpea genotypes. The figure on the left **(A)** represents the first principal component (PC1; x-axis) and the second principal compnent (PC2; y-axis), and the figure on the right **(B)** represents PC1 and the third principal component (PC3; y-axis). In both plots, genotypes are coloured according to chickpea type.

Population structure was investigated for the 148 genotypes and filtered marker set, principal component analysis (PCA) and phylogenetic cluster dendrogram were used to assess genetic relationships between individuals. Kinship matrix was calculated using the “synbreed” package in R (Wimmer et al., 2012), and visualised as a PCA using ggplot2 (Wickham, 2016) with genotypes colour coded by Desi and Kabuli type chickpeas (**Appendix 1**, **Figure 1**).

### 2.7 Genome wide association analysis and marker projection

GWAS analysis was used to identify QTL, which refers to an identifiable genomic region that can be associated with the presentation of a particular phenotype (Abiola et al., 2003). GWAS analysis was conducted using the “rrBLUP” package (Endelman, 2011), this package implements the unified mixed-model approach of Yu et al. (2006). Phenotypic data was reasonably normally distributed therefore transformations did not result in any significant changes in the association results. This stability suggests that the rrBLUP model effectively managed deviations from normality and other potential biases in the raw data. Four approaches were trialled, and the most appropriate model selected and implemented. Models tested include: i) naïve model with no measures of relatedness, ii) K model, incorporating the kinship structure, iii) QK model, building on relatedness by chickpea type and origin as a measure of population structure, and iv) QK model using Principal Components (PCs) as a measure of population structure. Final model selected was a QK fixed for chickpea type and including 3 PCs. QQ plots were generated for the -log10 transformed p-values derived from the association tests performed on each phenotypic trait (**Appendix 3**). Deviations of the points from the red line indicate potential genetic associations with the trait. Upward deviations, particularly at the tail end, suggest the presence of true genetic signals, while flatter regions near the middle indicate a good fit to the null hypothesis. Markers associated with traits at significance level of −log10p ≥ 3 were referred to as MTAs (marker-trait associations), regions with >1 significant marker within 10mbp were considered putative QTL. Manhattan plots were created by plotting the -log10(p) values of the marker-trait associations against their chromosomal positions using the R package ‘qqman’ (Turner, 2018). Genome-wide significance thresholds were set at -log10(p) = 3 (**Supplementary Figure 3**). Marker effects were estimated utilising the GWASPoly package (Rosyara et al., 2016). Visualisation was performed using Mapchart 2.32 (Voorrips, 2002). Known genes and QTL were located from existing literature, and their sequences identified using the NCBI Basic Local Alignment Search Tool (BLAST) (Sayers et al., 2022). These genes were added to the same map to determine validity of data and existence of novel QTL. Novel QTL were named according to their identified chromosome, their order, and the physiological trait associated. In total, 48 GWAS (148 individuals, across 2 environments, 3 years, 2 TOS, and 5 traits, minus 12 missing environments in **Table 1**) were conducted. From this, 56 significant MTA were identified, contributing to 14 QTL identified across 4 chromosomes. QTL were defined within each analysis, leading to different trials producing different QTL.

## 3 Results

### 3.1 Genotypic and phenotypic diversity

Principal component analysis PC1 and PC2 explained the accumulated genotypic variation of 20.50% and 8.93%, respectively (**Figure 1**). Genotypic marker data, clustered by chickpea type (Desi and Kabuli), there was sufficient overlap to allow interbreeding among genotypes, and the population has suitable diversity to mine for QTL. Along with this genetic variation, there was a strong relationship between environment and trait performance, demonstrated by the variation among all treatment levels (Year, TOS, Environment) and across all traits (**Table 1**), while phenotypic data remained within the limits of normal distribution.

### 3.2 Novel and validated QTL identified by this study

**Figure 2:**
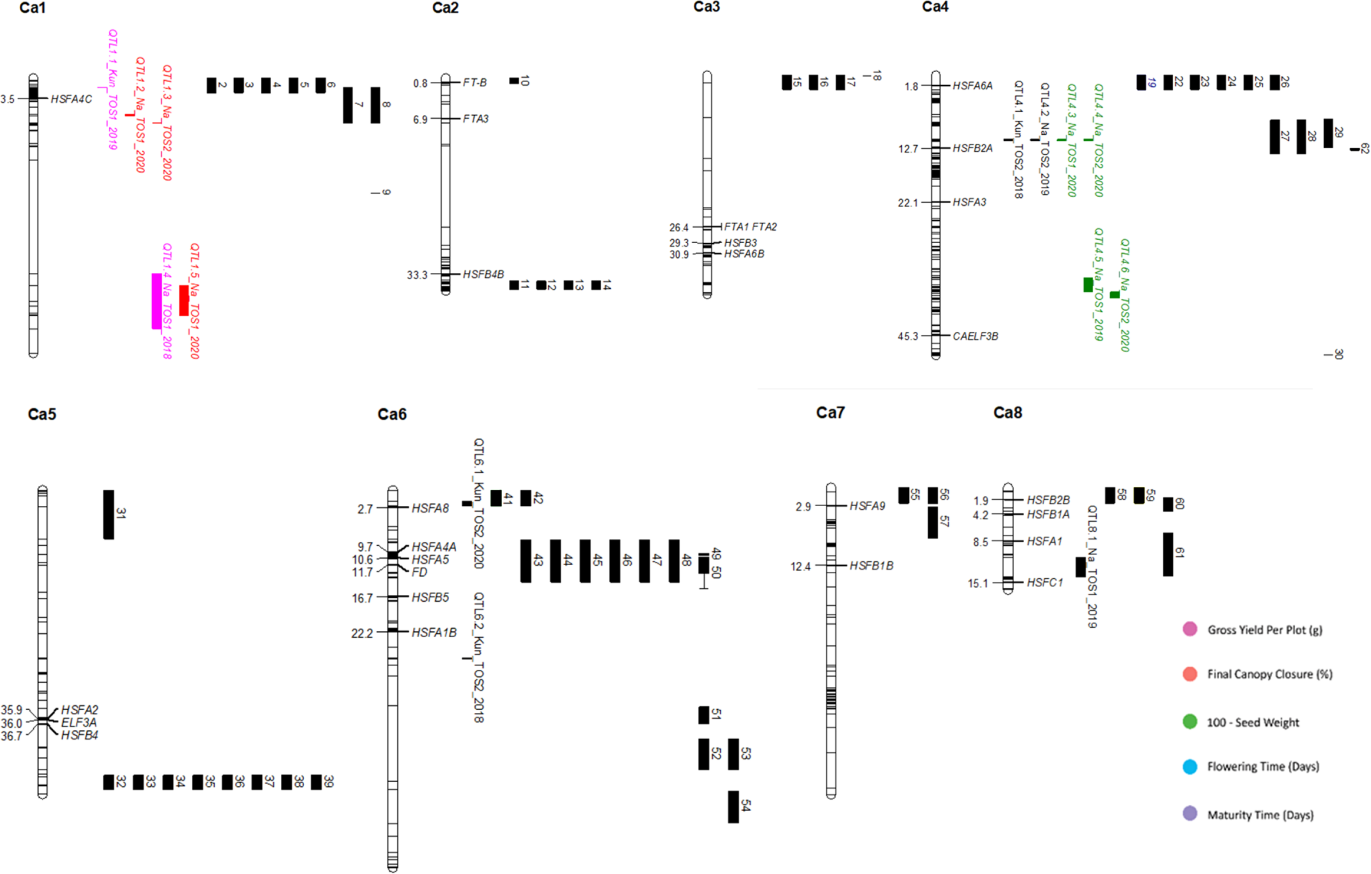
Physical map of QTLs identified in this study, along with the location of relevant and related gene and QTL findings from previous research. Chromosomes are represented as white vertical lines, with mapping markers denoted by black lines across the chromosomes. Genes are positioned according to Mb, and external QTLs are represented by black bands, numbered in order of chromosome and position, to the right of their relevant chromosome. QTLs identified by this study are represented by coloured bands, and labelled according to their linked trait. Positions are based on the CDC Frontier genome assembly (BioProject ID: PRJNA175619, NCBI Reference: NC_011163.1). A complete list of genes and QTLs, including their original position formats can be found in **Supplementary Data Sheet 1**. Colours denote putative roles in the following traits: Pink, total yield per plot (grams); Orange, final canopy closure (percentage); Green, 100-seed weight; Blue, days to 50% plot flowering; Purple, days to 50% maturation.

This study identified a total of 14 QTL across 8 genomic regions, relating to gross yield, HSW, flowering age, maturity age, and final canopy closure. The QTL were located on chromosomes 1, 4, 6 and 8 (**Table 2**; **Figure 2**). Five QTL, one for each trait; were novel, located in regions that have not previously been linked to traits of interest, two of which were co-located (**Figure 2**). For each of the measured traits, there was at least 1 QTL aligning with genetic region associated with previously described QTL (**Figure 2**). QTL for flowering are the most extensively reported in literature (**Table 3**), whereas for this study, the trait with the highest representation of QTL was HSW (**Figure 2**, **Table 2**). Most QTL were related to the Narrabri field trials (10 of 14) particularly TOS1 (6), conversely there were more linked to the Kununurra TOS2 trials (3) than TOS1 (1) (**Table 2**). QTL associated with yield were detected in TOS1 trials, one at each location, whereas those for HSW span multiple trials (**Table 2**).

**Table 2:** QTLs identified via GWAS for five physiological traits across 147 genotypes in 12 trials.

Ch	QTL Name	Trait	Linked Trial	# of Associated Markers	Marker Effect	Left Location	Right Location
Ca1	Qyield_1.1	Gross Yield Per Plot	Kun_2019_TOS1	2	-228.14	1.744678	1.744744
	Qcanopy_1.1	Canopy Closure	Na_2020_TOS1	2	-4.42	6.161699	6.383597
	Qcanopy_1.2	Canopy Closure	Na_2020_TOS2	2	0.91	7.630859	7.649141
	Qyield_1.2	Gross Yield Per Plot	Na_2018_TOS1	11	-376.00	33.185604	42.615142
	Qcanopy_1.3	Canopy Closure	Na_2020_TOS1	8	-9.61	35.323486	40.351158
Ca4	Qhsw_4.1	Seed Size	Kun_2018_TOS2	5	-3.12	11.212337	11.441367
	Qhsw_4.2	Seed Size	Na_2019_TOS2	5	-2.39	11.212337	11.441367
	Qhsw_4.3	Seed Size	Na_2020_TOS1	5	-2.98	11.212337	11.441367
	Qhsw_4.4	Seed Size	Na_2020_TOS2	5	-3.00	11.212337	11.441367
	Qflo_4.1	Flowering Time	Na_2019_TOS1	3	-0.08	35.29603	37.725454
	Qmat_4.1	Maturity Time	Na_2020_TOS2	3	2.15	37.725454	38.784252
Ca6	Qflo_6.2	Flowering Time	Kun_2020_TOS2	2	-1.41	1.829402	2.53797
	Qhsw_6.5	Seed Size	Kun_2018_TOS2	2	-3.91	26.349028	26.360262
Ca8	Qmat_8.2	Maturity Time	Na_2019_TOS1	2	-1.07	11.150126	14.248237

**Table 3:** QTLs identified by external literature for chickpea, related to traits measured in this study.

Related To	QTL_ID In This Map	LG	QTL Name	Reference
Yield	2	Ca1	Q1-1	(Rehman et al., 2011)
	7		Qgy01_1	(Paul et al., 2018)
	8		Qgy01_1	
	21	Ca4	Qtlyd	(Cobos et al., 2007)
	26		Q4-1	(Rehman et al., 2011)
	36	Ca5	Qgy02_5	(Paul et al., 2018)
	37		Qgy02_5	
	47	Ca6	Qgy03_6	
	48		Qgy03_6	
	49		Casypp_NS6	(Jha et al., 2021)
Seed Size	20	Ca4	Qtlsw1	(Cobos et al., 2007)
	24			(Cho et al., 2002)
	42			(Jamalabadi et al., 2013)
	57	Ca7	Qph-2	(Gupta et al., 2015)
	62	Ca4	Ca4_TIFY4B	(Nguyen et al., 2022)
Seed Number/Plant	23	Ca4		(Cho et al., 2002)
Pod Setting %	27	Ca4	Q%Podset03_4	(Cobos et al., 2007; Paul et al., 2018)
	28		Q%Podset03_4	
	32	Ca5	Q%Podset06_5	
	33		Q%Podset06_5	
	43	Ca6	Q%Podset08_6	
	44		Q%Podset08_6	
Pod Filling	11	Ca2	Qfpod01_2	
	12		Qfpod01_2	
	34	Ca5	Qfpod02_5	
	35		Qfpod02_5	
	45	Ca6	Qfpod03_6	
	46		Qfpod03_6	
Number Of Seeds	13	Ca2	Qts01_2	
	14		Qts01_2	
	38	Ca5	Qts02_5	
	39		Qts02_5	
Number Of Filled Pods	51	Ca6	Cafp_NS6	(Jha et al., 2021)
Height	54	Ca3	Qsw-2	(Gupta et al., 2015)
	6	Ca1	Q1-1	(Rehman et al., 2011)
	25	Ca4	Q4-3	
Early Flowering	22	Ca4	QTLEF1	(Cobos et al., 2007)
Double Podding	41	Ca6		(Cho et al., 2002)
Days To Podding	9	Ca1	Cadpi_LS1	(Jha et al., 2021)
	53	Ca6	Cadpi_LS6	
Days To Pod Filling	30	Ca4	Cadpf_NS4	
	60	Ca8	Cadpf_LS8	
Days To Maturity	4	Ca1	Q1-1	(Rehman et al., 2011)
	16	Ca3	Q3-1	
	55	Ca7	Q7-1	
Days To Flowering	1	Ca1	Qefl2-1	(Mallikarjuna et al., 2017)
	3	Ca1	Q1-1	(Rehman et al., 2011)
	10	Ca3	Qefl1-1	(Jamalabadi et al., 2013)
	18		Qefl3-2	
	15	Ca3	Q3-1	(Rehman et al., 2011)
	19	Ca4	Qefl1-2	(Mallikarjuna et al., 2017)
	29		Qefl2-3	
	31	Ca3	Qefl3-1	
	40	Ca6	Qefl4-1	
	50	Ca1	QTL1	(Lichtenzveig et al., 2006)
	52	Ca6	Cadfi_LS6	(Jha et al., 2021)
	58	Ca8	Qefl2-4	(Mallikarjuna et al., 2017)
	59	Ca8	Q8-2	(Rehman et al., 2011)
	61	Ca8	Cadfi_LS8	(Jha et al., 2021)
Days From Flowering to Maturity	5	Ca1	Q1-1	(Rehman et al., 2011)
	17	Ca3	Q3-1	
	56	Ca7	Q7-1	

Several of QTL identified overlap with QTL and genes identified in previous research, such as the co-location of Qyield_1.1 and Q1-1 (Rehman et al., 2011). In total, there are 5 regions where QTL have overlapping positions with mapped QTL and genes found in previous literature. These are as follows: (1) Qyield_1.1, overlapping with QTL IDs 1-6, linked to days to flowering, days to maturity, days from flowering to maturity, and height ( (Rehman et al., 2011; Mallikarjuna et al., 2017), and HSFA4C (Chidambaranathan et al., 2018), (2) Qcanopy_1.1 and Qcanopy_1.2, overlapping with QTL IDs 7 and 8, linked to yield (Paul et al., 2018), (3) Qhsw_4.1-4.4, overlapping with QTL IDs 27-29, linked to pod setting and days to flowering (Cobos et al., 2007; Mallikarjuna et al., 2017), and HSFB2A (Chidambaranathan et al., 2018), (4) Qflo_6.2, overlapping with QTL IDs 41 and 42, linked to double podding and seed size (Cho et al., 2002; Jamalabadi et al., 2013), and HSFA8 (Chidambaranathan et al., 2018), (5) Qmat_8.2, overlapping with QTL ID 61 linked to days to flowering (Jha et al., 2021) (**Figure 2**, **Tables 2**–**4**).

**Table 4:** Genes identified by external literature for chickpea, related to heat stress.

Symbol	LG	Description	Gene ID	Reference
HSFA4C	Ca1	Heat Stress Transcription Factor A-4c	101515265	(Chidambaranathan et al., 2018)
HSFB4B	Ca2	Heat Stress Transcription Factor B-4b	101499841	
HSFA6B	Ca3	Heat Stress Transcription Factor A-6b	101499033	
HSFB3		Heat Stress Transcription Factor B-3	101502316	
HSFA3	Ca4	Heat Stress Transcription Factor A-3	101513159	
HSFA6A		Heat Stress Transcription Factor A-6a	101493622	
HSFB2A		Heat Stress Transcription Factor B-2a	101505110	
HSFA2	Ca5	Heat Stress Transcription Factor A2	101510605	
HSFB4		Heat Stress Transcription Factor B-4	101514043	
HSFA1B	Ca6	Heat Stress Transcription Factor A-1b	101514812	
HSFA4A		Heat Stress Transcription Factor A-4a	101489852	
HSFA5		Heat Stress Transcription Factor A-5	101494390	
HSFA8		Heat Stress Transcription Factor A-8	101489530	
HSFB5		Heat Stress Transcription Factor B-5	101504520	
HSFA9	Ca7	Heat Stree Transcription Factor A-9	101495918	
HSFB1B		Heat Stree Transcription Factor B-1b	101504213	
HSFA1	Ca8	Heat Stree Transcription Factor A-1	101492154	
HSFB1A		Heat Stress Transcription Factor B-1a	101494022	
HSFB2B		Heat Stress Transcription Factor B-2b	101502512	
HSFC1		Heat Stress Transcription Factor C-1	101489265	
FTA3	Ca2	Protein FLOWERING LOCUS T	101515383	(Ridge et al., 2017)
FT-B		Protein FLOWERING LOCUS T	101505276	
FTA1	Ca3	Protein FLOWERING LOCUS T	101497376	
FTA2		Protein FLOWERING LOCUS T-Like	101496618	
CAELF3B	Ca4	Protein EARLY FLOWERING 3b	101488316	
ELF3A	Ca5	Protein EARLY FLOWERING 3a	101489432	
FD	Ca6	Protein FLOWERING LOCUS D	101490188	

## 4 Discussion

The QTL identified have the potential to advance current knowledge of the chickpea genome. The overlap of new and previously discovered QTL on chromosomes 1, 4, 6 and 8 suggest the presence of highly valuable QTL hotspots, which could be targeted for marker-assisted breeding for future improvement of the crop (**Figure 2**, **Tables 2**, **3**). This is particularly exciting, considering the range of traits these QTL are associated with.

Due to the large number of trials on which these findings are based, it is also possible to infer and predict the expression of each QTL, based on the presence of a stressor. This provides a deeper understanding of the plasticity of these traits, and improved understanding of the expression of these traits under particular conditions. Most QTL found in this study are linked to Narrabri field trials, particularly those of TOS1, and these are linked both to physiological and yield traits (**Table 2**). This may be related to the domestication of chickpeas to a Mediterranean environment, which is similar in climate to Narrabri (Kumar and Abbo, 2001), reflecting selection pressure for this environment type. However, the location of Qflo_6.2, linked to flowering time in Kununurra TOS2 2020, indicates the expression of a genomic region linked to flowering under this particular heat stress, given that it does not co-locate with other mapped QTL for flowering time (Lichtenzveig et al., 2006) (**Figure 2**, **Table 3**). Qflo_6.2 overlaps with the mapped gene HSFA8, which is a HSF linked to shoot apical meristem and floral tissue development under heat stress (Chidambaranathan et al., 2018) (**Figure 2**, **Table 3**). This overlap suggests that Qflo_6.2 is linked to a developmental acceleration under heat stress, particularly as early flowering, or “escape”, is a known physiological strategy for coping with heat stress (Kumar and Abbo, 2001), and plant growth was accelerated under the higher temperatures in Kununurra. This is further suggested by the presence of the other QTL for flowering time, Qflo4.1, which was identified in Narrabri TOS1 2019, overlapping with Qmat_4.1 (**Figure 2**, **Table 2**). This could be caused by differential protein expression that is dependent on the environmental conditions. In contrast, the QTL identified for HSW predominately share an overlapping location on chromosome 4, though they are linked to different trials (**Figure 2**). This suggests that the expression of the genomic region responsible for seed size is not strongly impacted by environment, making it desirable for genomic selection (Pierce, 2020). This is supported by an investigation by Jeffrey et al. (under review)^1^, which found HSW to be highly heritable, particularly compared to yield. Additionally, the overlap of Qhsw_4.1-4.4 and the gene HSFB2A may indicate that under heat stress, plants divert energy away from new growth and into existing structures, such as seeds. HSFB2A is an HSF that is upregulated under heat stress, linked to the development of all tissue types except those of shoots, and linked most heavily with the development of young pods (Chidambaranathan et al., 2018). Similarly, the QTL associated with yield in this study were both linked to TOS1 trials, although different locations, suggesting that yield may be more heritable under stress-free conditions (**Figure 2**, **Table 2**). This suggestion validates the need for lab-based markers related to yield in this species, as it would assist breeders in marker assisted selection (MAS).

### 4.1 Canopy closure – A recommendation for future research

This study is the first to describe genomic regions associated with final canopy closure in chickpea. This is an exciting discovery both from the perspective of novel QTL discovery, and the potential to build on research that is focused on stress management. Much previous research has focused on heat escape, examining traits such as earliness in flowering and maturity (Berger et al., 2011). However, while breeding for heat escape can be beneficial, this is only a solution if seasons remain predictable, and temperature shifts minimal, which does not align with recent predictions (NASA, 2022). It has been known for some time that canopy climate is crucial for the maintenance of healthy plant tissues, particularly reproductive organs (Gates, 1968). The creation of shade via canopy closure has the potential to protect root systems, and their rhizobial symbionts, from overheating (Lewis et al., 2000). Additionally, as effective transpiration can cool leaves from 6-10°C, canopy closure can create a cooler zone in which pods and other delicate tissues can grow with reduced heat stress (Mathur et al., 2014). This study identified 3 novel QTL linked to final canopy closure, which are also linked to yield QTL found by both this and previous research (Paul et al., 2018; Jha et al., 2021). This doesn’t just give the potential to produce new chickpea genotypes that survive under high temperature, but also the potential to create genotypes that can manage their own stress response and thrive through physiological processes, without sacrificing yield. Finally, Qyield_1.1, Qcanopy_1.1, and Qcanopy_1.2 overlap with a QTL for flowering date (Mallikarjuna et al., 2017), a QTL for yield, flowering time, height, and maturity (Rehman et al., 2011), and a QTL for yield (Paul et al., 2018), along with gene HSFA4C, which is linked to a heat stress transcription factor (HSF), and expressed in the presence of a *fusarium* wilt infection (Chidambaranathan et al., 2018) (**Figure 2**, **Tables 2**, **3**). This overlap suggests that these particular traits are linked or pleiotropic, suggesting the potential for GAB to vastly improve the performance of future chickpea genotypes (Pierce, 2020).

The discovery of QTL linked to this trait creates the potential for further understanding of its expression, control, and breeding potential, and this could lead to the development of genotypes that can respond to and manage stress at a significantly more efficient and effective rate. Additionally, as transpiration efficiency has been found to have a heritability rate of up to 70% (Thudi et al., 2014), and stomatal conductance is seen to be higher in heat tolerance genotypes (Kaushal et al., 2013), breeding plans can be created that target all of these traits, enhancing the tools we currently have for breeding.

Based on the review of existing literature (**Table 3**), this study identified the first QTL associated with final canopy closure in Chickpea, with Qcanopy_1.1, 1.2, and 1.3 identified on chromosome 1, and closely linked to yield-linked QTL Qyield_1.1 and 1.2, and those discovered in previous literature designated QTL 7 and 8, also linked to yield (Paul et al., 2018) (**Figure 2**, **Table 2**). These QTL were found across both trials in which the trait was measured (**Table 2**).

## Data Availability

Data is available within **Supplementary Data Sheet 1** and at NCBI with accession number: 14822344.

## Footnotes

1 Jeffrey, C., Ziems, L., Kaiser, B., and Trethowan, R. A Growing Degree Day (GDD) model determines the effect of temperature stress on diverse chickpea genotypes. *Front. Plant Sci.* (under review).

## References

[B1] Abbo S. Berger J. Turner N. C. (2003). Evolution of cultivated chickpea: four bottlenecks limit diversity and constrain adaptation. Funct. Plant Biol. 30, 1081–1087. doi: 10.1071/FP03084 32689090

[B2] Abiola O. Angel J. M. Avner P. Bachmanov A. A. Belknap J. K. Bennett B. (2003). The nature and identification of quantitative trait loci: a community's view. Nat. Rev. Genet. 4, 911–916. doi: 10.1038/nrg1206 14634638 PMC2063446

[B3] Ahmad F. Slinkard A. (2004). The extent of embryo and endosperm growth following interspecific hybridization between *Cicer arietinum* L. and related annual wild species. Genet. Resour. - Crop Evol. 51, 765–772. doi: 10.1023/B:GRES.0000034580.67728.e4

[B4] Alonge M. Shumate A. Puiu D. Zimin A. V. Salzberg S. L. (2020). Chromosome-scale assembly of the bread wheat genome reveals thousands of additional gene copies. Genetics 216, 599–608. doi: 10.1534/genetics.120.303501 32796007 PMC7536849

[B5] Berger J. Milroy S. Turner N. Siddique K. Imtiaz M. Malhotra R. (2011). Chickpea evolution has selected for contrasting phenological mechanisms among different habitats. Euphytica 180, 1–15. doi: 10.1007/s10681-011-0391-4

[B6] Chidambaranathan P. Jagannadham P. T. K. Satheesh V. Kohli D. (2018). Genome-wide analysis identifies chickpea (*Cicer arietinum*) heat stress transcription factors (Hsfs) responsive to heat stress at the pod development stage. J. Plant Res. 131, 525–542. doi: 10.1007/s10265-017-0948-y 28474118

[B7] Cho S. Kumar J. Shultz J. L. Anupama K. Tefera F. Muehlbauer F. J. J. E. (2002). Mapping genes for double podding and other morphological traits in chickpea. Euphytica. 128, 285–292. doi: 10.1023/A:1020872009306

[B8] Cobos M. J. Rubio J. Fernández-Romero M. D. Garza R. Moreno M. T. Millán T.. (2007). Genetic analysis of seed size, yield and days to flowering in a chickpea recombinant inbred line population derived from a Kabuli × Desi cross. Annals of Applied Biology. 151, 33–42. doi: 10.1111/j.1744-7348.2007.00152.x

[B9] Devasirvatham V. Tan D. K. Gaur P. M. Trethowan R. M. (2015). Chickpea and temperature stress: An overview. Legumes under Environ. Stress: Yield Improve. Adaptations, 81–90. doi: 10.1002/9781118917091.ch5

[B10] Endelman J. (2011). Ridge regression and other kernels for genomic selection with R package rrBLUP. Plant Genome. doi: 10.3835/plantgenome2011.08.0024

[B11] FAO F. (2020). Crops (Rome, Italy: Food and Agriculture Organisation of the United Nations).

[B12] Farahani S. Maleki M. Mehrabi R. Kanouni H. (2019). Whole genome diversity, population structure, and linkage disequilibrium analysis of chickpea (*Cicer arietinum* L.) genotypes using genome-wide DArTseq-based SNP markers. Genes 10, 676. 31487948 10.3390/genes10090676 PMC6770975

[B13] Gates D. M. (1968). Transpiration and leaf temperature. Annu. Rev. Plant Physiol. 19, 211–238. doi: 10.1146/annurev.pp.19.060168.001235

[B14] Gupta S. Kumar T. Verma S. Bharadwaj C. Bhatia S. (2015). Development of gene-based markers for use in construction of the chickpea (Cicer arietinum L.) genetic linkage map and identification of QTLs associated with seed weight and plant height. Molecular biology reports 42, 1571–1580. 26446030 10.1007/s11033-015-3925-3

[B15] Hiremath P. J. Kumar A. Penmetsa R. V. Farmer A. Schlueter J. A. (2012). Large-scale development of cost-effective SNP marker assays for diversity assessment and genetic mapping in chickpea and comparative mapping in legumes. Plant Biotechnol. J. 10, 716–732. doi: 10.1111/j.1467-7652.2012.00710.x 22703242 PMC3465799

[B16] IBPGR, I (1993). ICARDA, Descriptor for chickpea (Cicer arietinum L.) (Rome Italy: IBPGR).

[B17] IPCC (2021). “Climate Change 2021: The Physical Science Basis,” in Contribution of Working Group I to the Sixth Assessment Report of the Intergovernmental Panel on Climate Change. Eds. Arias P. A. Bellouin N. Coppola E. Jones R. G. Krinner G. Marotzke J. Naik V. Palmer M. D. Plattner G. K. Rogelj J. Rojas M. Sillmann J. Storelvmo T. Thorne P. W. Trewin B. Rao K.A. Adhikary B. Allan R. P. Armour K. Bala G. Barimalala R. Berger S. Canadell J. G. Cassou C. Cherchi A. Collins W. Collins W. D. Connors S. L. Corti S. Cruz F. Dentener F. J. Dereczynski C. Luca A. Niang A.D. Doblas-Reyes F. J. Dosio A. Douville H. Engelbrecht F. Eyring V. Fischer E. Forster P. Fox-Kemper B. Fuglestvedt J. S. Fyfe J. C. Gillett N. P. Goldfarb L. Gorodetskaya I. Gutierrez J. M. Hamdi R. Hawkins E. Hewitt H. T. Hope P. Islam A. S. Jones C. Kaufman D. S. Kopp R. E. Kosaka Y. Kossin J. Krakovska S. Lee J. Y. Li J. Mauritsen T. Maycock T. K. Meinshausen M. Min S. K. Monteiro P. M. S. Ngo-Duc T. Otto F. Pinto I. Pirani A. Raghavan K. Ranasinghe R. Ruane A. C. Ruiz L. Sallée J. B. Samset B. H. Sathyendranath S. Seneviratne S. I. Sörensson A. A. Szopa S. Takayabu I. Tréguier A.-M. Hurk B.V. D. Vautard R. Schuckmann K.V. Zaehle S.(Cambridge, United Kingdom, and New York, NY, USA: The IPCC Secretariat).

[B18] Jamalabadi J. G. Saidi A. Karami E. Kharkesh M. Talebi R. (2013). Molecular mapping and characterization of genes governing time to flowering, seed weight, and plant height in an intraspecific genetic linkage map of chickpea (*Cicer arietinum*). Biochem. Genet. 51, 387–397. doi: 10.1007/s10528-013-9571-3 23371372

[B19] Jeffrey C. Trethowan R. Kaiser B. (2021). Chickpea tolerance to temperature stress: Status and opportunity for improvement. J. Plant Physiol. 267, 153555. doi: 10.1016/j.jplph.2021.153555 34739858

[B20] Jha U. C. Nayyar H. Palakurthi R. Jha R. (2021). Major QTLs and potential candidate genes for heat stress tolerance identified in chickpea (*Cicer arietinum* L.). Front. Plant Sci. 12, 655103. doi: 10.3389/fpls.2021.655103 34381469 PMC8350164

[B21] Kaushal N. Awasthi R. Gupta K. Gaur P. (2013). Heat-stress-induced reproductive failures in chickpea (*Cicer arietinum*) are associated with impaired sucrose metabolism in leaves and anthers. Funct. Plant Biol. 40, 1334–1349. doi: 10.1071/FP13082 32481199

[B22] Kumar J. Abbo S. (2001). “Genetics of flowering time in chickpea and its bearing on productivity in semiarid environments,” in Advances in Agronomy (Basel, Switzerland: International Crops Research Institute for the Semi Arid Tropics).

[B23] Kumar S. Thakur P. Kaushal N. Malik J. A. Gaur P. Nayyar H. (2013). Effect of varying high temperatures during reproductive growth on reproductive function, oxidative stress and seed yield in chickpea genotypes differing in heat sensitivity. Arch. Agron. Soil Sci. 59, 823–843. doi: 10.1080/03650340.2012.683424

[B24] Lewis C. E. Noctor G. Causton D. Foyer C. H. (2000). Regulation of assimilate partitioning in leaves. Funct. Plant Biol. 27, 507–519. doi: 10.1071/PP99177

[B25] Lichtenzveig J. Bonfil D. J. Zhang H.-B. Shtienberg D. Abbo S. (2006). Mapping quantitative trait loci in chickpea associated with time to flowering and resistance to Didymella rabiei the causal agent of Ascochyta blight. Theor. Appl. Genet. 113, 1357–1369. doi: 10.1007/s00122-006-0390-3 17016689

[B26] Mallikarjuna B. P. Samineni S. Thudi M. Sajja S. B. (2017). Molecular mapping of flowering time major genes and QTLs in chickpea (*Cicer arietinum* L.). Front. Plant Sci. 8, 1140. doi: 10.3389/fpls.2017.01140 28729871 PMC5498527

[B27] Maphosa L. Richards M. F. Norton S. L. Nguyen G. N. (2020). Breeding for abiotic stress adaptation in chickpea (Cicer arietinum L.): A comprehensive review. Crop Breed. Genet. Genomics 4, 1–39. doi: 10.20900/cbgg20200015

[B28] Mathur S. Agrawal D. Jajoo A. (2014). Photosynthesis: Response to high temperature stress. J. Photochem. Photobiol. B: Biol. 137, 116–126. doi: 10.1016/j.jphotobiol.2014.01.010 24796250

[B29] NASA (2022). Global Climate Change Impact on Crops Expected Within 10 Years. Available online at: https://climate.nasa.gov/news/3124/global-climate-change-impact-on-crops-expected-within-10-years-nasa-study-finds/:~:text=en%20espa%C3%B1ol%20aqui.-,Climate%20change%20may%20affect%20the%20production%20of%20maize%20(corn)%20and,see%20growth%20of%20about%2017%25 (accessed October 1, 2022).

[B30] Nguyen D. T. Hayes J. E. Harris J. Sutton T. (2022). Fine mapping of a vigor QTL in chickpea (Cicer arietinum L.) reveals a potential role for Ca4_TIFY4B in regulating leaf and seed size 13, 829566. doi: 10.3389/fpls.2022.829566 PMC8908238 35283931

[B31] Pandey M. K. Roorkiwal M. Singh V. K. Ramalingam A. Kudapa H. (2016). Emerging genomic tools for legume breeding: current status and future prospects. Front. Plant Sci. 7, 1–18. doi: 10.3389/fpls.2016.00455 27199998 PMC4852475

[B32] Patrignani A. Ochsner T. E. (2015). Canopeo: A powerful new tool for measuring fractional green canopy cover. Biometry Model. Stat 107, 2312–2320. doi: 10.2134/agronj15.0150

[B33] Paul P. J. Samineni S. Thudi M. Sajja S. B. Rathore A. (2018). Molecular mapping of QTLs for heat tolerance in chickpea. Int. J. Mol. Sci. 19, 2166. doi: 10.3390/ijms19082166 30044369 PMC6121679

[B34] Pierce B. A. (2020). Genetics: a conceptual approach. 7th edn (New York, USA: WH Freeman New York).

[B35] Pundir R. Reddy K. Mengesha M. H. (1988). ICRISAT chickpea germplasm catalog: evaluation and analysis (India: International Crops Research Institute for the Semi-Arid Tropics).

[B36] Rehman A. U. Malhotra R. S. Bett K. Tar'an B. (2011). Mapping QTL Associated with Traits Affecting Grain Yield in Chickpea (*Cicer arietinum* L.) under Terminal Drought Stress. Crop Science. 51, 450–463. doi: 10.2135/cropsci2010.03.0129

[B37] Ridge S. Deokar A. Lee R. Daba K. Macknight R. C. Weller J. L.. (2017). The chickpea Early Flowering 1 (Efl1) locus is an ortholog of Arabidopsis ELF3. Plant physiology 175 (2), 802–815. 28818860 10.1104/pp.17.00082 PMC5619881

[B38] Roorkiwal M. Rathore A. Das R. R. Singh M. K. Jain A. (2016). Genome-enabled prediction models for yield related traits in chickpea. Front. Plant Sci. 7, 1666. doi: 10.3389/fpls.2016.01666 27920780 PMC5118446

[B39] Rosyara U. R. De Jong W. S. Douches D. S. Endelman J. B. (2016). Software for genome-wide association studies in autopolyploids and its application to potato. PlantGenome 9, 2. doi: 10.3835/plantgenome2015.08.0073 27898814

[B40] Sayers E. W. Bolton E. E. Brister J. R. Canese K. Chan J. Comeau D. C.. (2022). Database resources of the national center for biotechnology information. Nucleic Acids Res. 50, D20–d26. doi: 10.1093/nar/gkab1112 34850941 PMC8728269

[B41] Sharma S. Upadhyaya H. D. Roorkiwal M. Varshney R. K. Gowda C. L. L. (2013). “4 - Chickpea,” in Genetic and Genomic Resources of Grain Legume Improvement. Eds. Singh M. Upadhyaya H. D. Bisht I. S. (Elsevier, Oxford).

[B42] Thudi M. Upadhyaya H. D. Rathore A. Gaur P. M. Krishnamurthy L. Roorkiwal M.. (2014). Genetic dissection of drought and heat tolerance in chickpea through genome-wide and candidate gene-based association mapping approaches. PloS One 9, e96758. doi: 10.1371/journal.pone.0096758 24801366 PMC4011848

[B43] Turner S. (2018). qqman: an R package for visualizing GWAS results using QQ and Manhattan plots. J. Open Source Softw. 3, 731. doi: 10.1101/005165

[B44] Upadhyaya H. D. Dronavalli N. Gowda C. Singh S. (2011). Identification and evaluation of chickpea germplasm for tolerance to heat stress. Crop Sci. 51, 2079–2094. doi: 10.2135/cropsci2011.01.0018

[B45] Varshney R. K. Song C. Saxena R. K. Azam S. (2013). Draft genome sequence of chickpea (*Cicer arietinum*) provides a resource for trait improvement. Nat. Biotechnol. 31, 240. doi: 10.1038/nbt.2491 23354103

[B46] Varshney R. K. Thudi M. Muehlbauer F. J. (2017). “The Chickpea Genome: An Introduction,” in The Chickpea Genome. Eds. Varshney R. K. Thudi M. Muehlbauer F. (Springer International Publishing, Cham).

[B47] Voorrips R. E. (2002). MapChart: Software for the graphical presentation of linkage maps and QTLs. J. Hered. 93, 77–78. doi: 10.1093/jhered/93.1.77 12011185

[B48] VSN-International (2022). Genstat for Windows 22nd Edition (Hemel Hempstead, UK: VSN International).

[B49] Wickham H. (2016). ggplot2: Elegant Graphics for Data Analysis (New York USA: Springer-Verlag).

[B50] Wimmer V. Albrecht T. Auinger Hj. Schoen Cc. (2012). synbreed: a framework for the analysis of genomic prediction data using R. Bioinformatics. 28, 2086–2087. doi: 10.1093/bioinformatics/bts335 22689388

[B51] Winter P. Benko-Iseppon A.-M. Hüttel B. Ratnaparkhe M. (2000). A linkage map of the chickpea (*Cicer arietinum* L.) genome based on recombinant inbred lines from a C. arietinum× C. reticulatum cross: localization of resistance genes for fusarium wilt races 4 and 5. Theor. Appl. Genet. 101, 1155–1163. doi: 10.1007/s001220051592

[B52] Yu J. Pressoir G. Briggs W. H. Bi I. V. Yamasaki M. Doebley J. F.. (2006). A unified mixed-modelmethod for association mapping that accounts for multiple levels of relatedness. Nat. Genet. 38, 203–208. doi: 10.1038/ng1702 16380716

