# Supplementary figures and images for: Genome-wide association study reveals heat tolerance QTL for canopy-closure and early flowering in chickpea
Source: Front Plant Sci. 2024 Dec 17;15:1458250. doi: 10.3389/fpls.2024.1458250 (PMC11685022; doi:10.3389/fpls.2024.1458250)

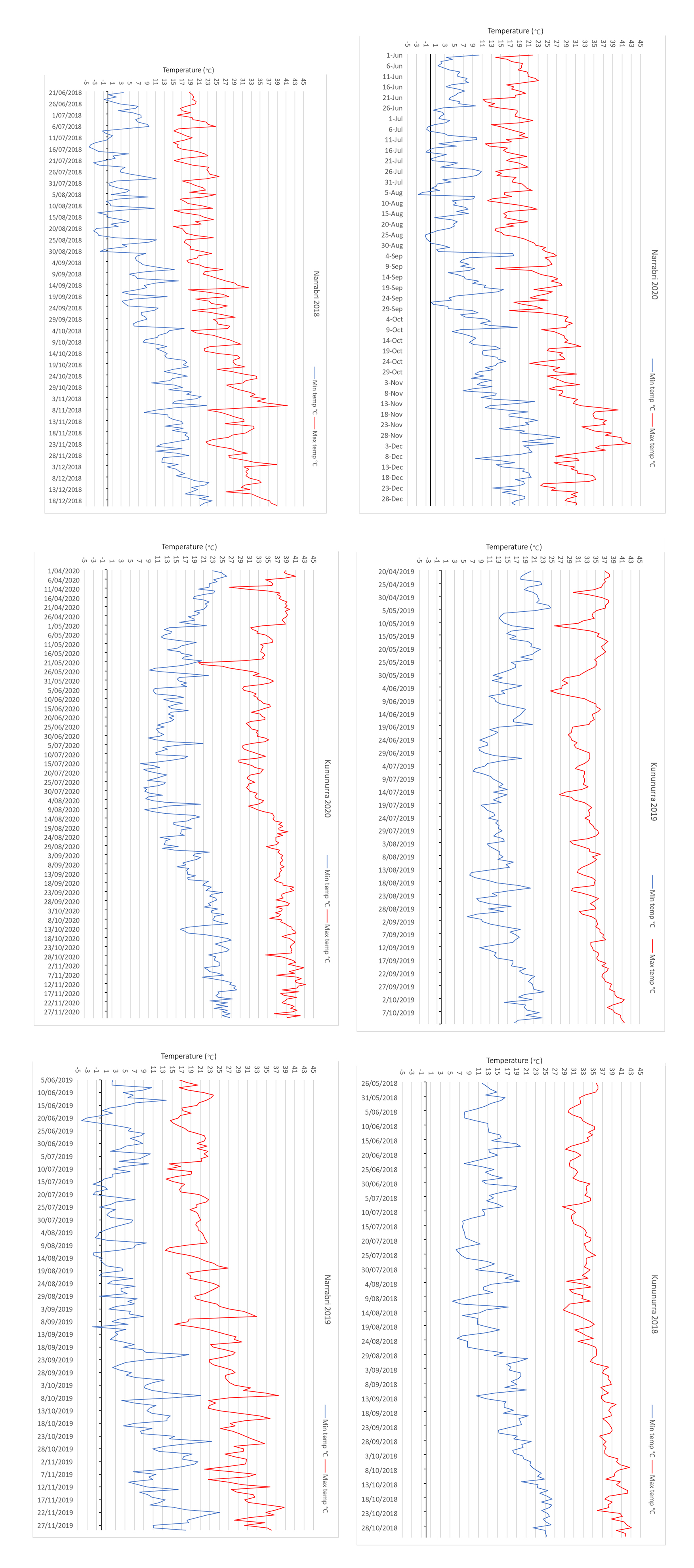

Supplement: Supplementary file 2 [file Image1.jpg]

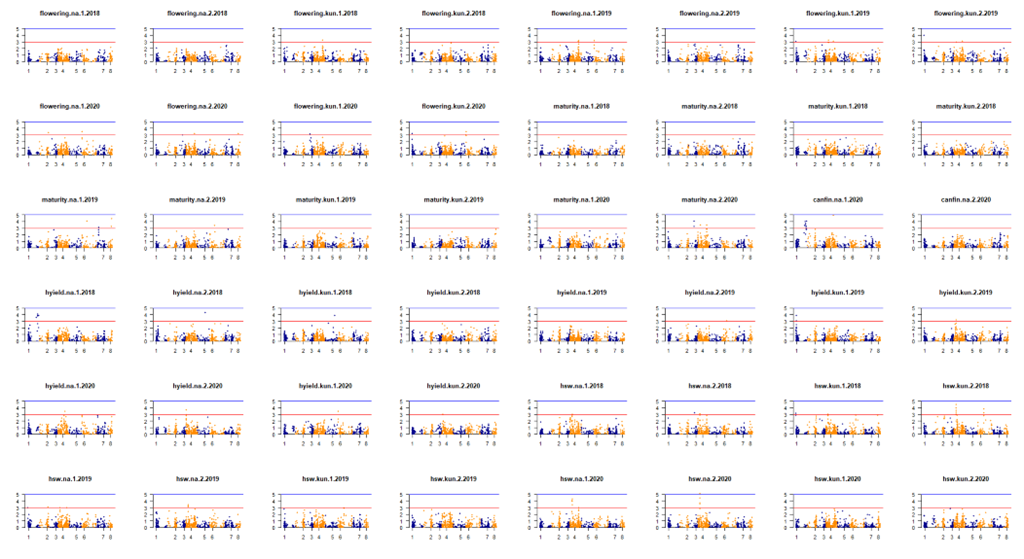

Supplement: Supplementary file 3 [file Image2.tif]

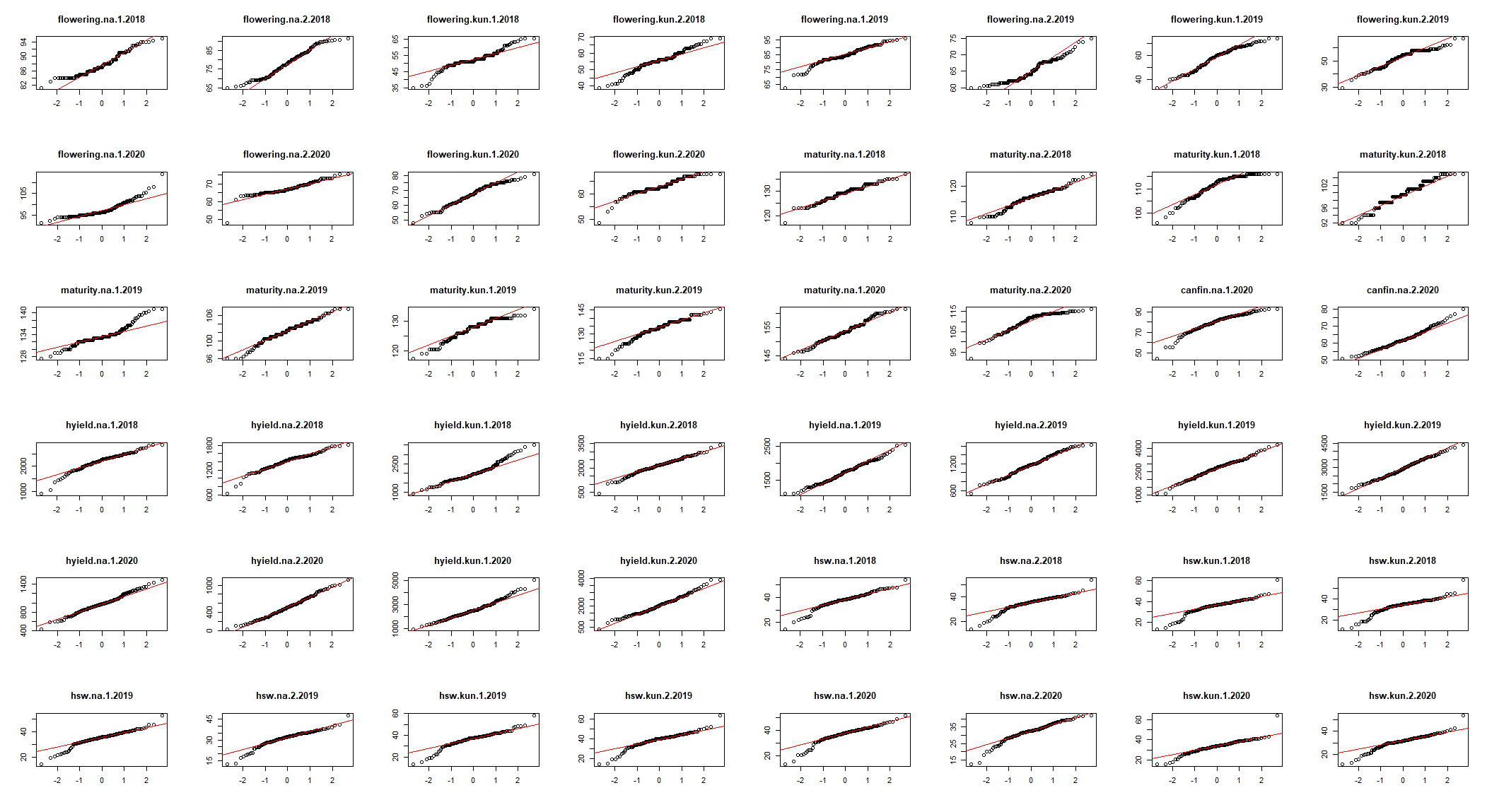

Supplement: Supplementary file 4 [file Image3.tiff]
